# 58 Comparison of the 2015 pediatric diagnostic criteria for Behcet’s disease with the 2014 and 1990 International Study Group Diagnostic Criteria

**DOI:** 10.1093/rheumatology/keac496.054

**Published:** 2022-10-07

**Authors:** K El Ouassifi, A Sakhi, K Bouayed, S Zoukal, S Nani

**Affiliations:** 1 Department of Internal Medicine and Pediatric Rheumatology, A. Harouchi, Mother-Child Hospital, Casablanca, Morocco; 2 Epidemiology Department, Faculty of Medicine and Pharmacy, Casablanca, Morocco

## Abstract

**Background:**

Behçet's disease is a multisystem vasculitis whose pathogenesis remains unclear. Although usually described in young adults, it may begin in childhood. The diagnosis is clinical, based on international criteria. The limitations of early diagnosis are related to the progressive onset of symptoms and the variety of differential diagnoses at this age in the absence of a pathognomonic diagnostic test.

**Objectives:**

To report the epidemiological features of our series and to compare the 2015 pediatric criteria with the 2014 and 1990 international criteria.

**Materials and Methods:**

This is a monocentric, retrospective study of 31 children over an 11-year period from January 2011 to December 2021. All patients suspected with Behcet's disease by a pediatric rheumatologist were included in the study, the 2014 international criteria “ISG 2014” being the gold standard classification criteria used in our center.

**Results:**

31 cases of Behcet's were collected. The mean age was 10 years (5.5–16) ± 2.87 years. A female predominance is noted with a sex ratio F/M of 1.21. A quarter of the patients were from a consanguineous marriage and 38.7% had a family history of Behcet disease. Mucocutaneous manifestations were represented by recurrent oral aphthosis 87.1%, genital aphthosis 29%, pseudofolliculitis 48.4%, erythema nodosum 6.5% and acne lesions 3.2%. The pathergy test was positive in 1 case. Ocular involvement was reported in 29% and joint involvement in 45.2%. Thromboembolic complication was seen in 9.6% and neurobehcet in one patient. The HLA B51 antigen was present in 45.2% of cases. In our series 38.7% respond the pediatric criteria, while 61.3% met the 2014 international criteria and 35.5% met the 1990 international criteria. The 2015 pediatric criteria have respectively a sensitivity and specificity of 63.2% and 100% (*p* = 0.002) compared with the 2014 international criteria taken as gold standard, as well as the 1990 international criteria with a respective sensitivity and specificity of 52.6% and 91.7% (*p* = 0.034).

**Conclusion:**

Our study highlights a female predominance, a high rate of consanguinity and familial Behcet. The sensitivity and specificity of the 1990 pediatric and international criteria appear to be better than those of 2014, with a more significant trend for the 2015 pediatric criteria (*p* = 0.002).

